# Network meta-analysis of the intervention effects of different exercise measures on Sarcopenia in cancer patients

**DOI:** 10.1186/s12889-024-18493-y

**Published:** 2024-05-10

**Authors:** Rui Liu, XY Gao, Li Wang

**Affiliations:** Department of Special Medical Care, Cancer Hospital, Chinese Academy of Medical Sciences, Chaoyang district, 100021 Beijing, China

**Keywords:** Tumour, Sarcopenia, Exercise intervention, Network Meta-analysis

## Abstract

**Purpose:**

This study aims to investigate the impact of four exercise modes (aerobic exercise, resistance exercise, aerobic combined with resistance multimodal exercise, and stretching) on the physical performance of cancer patients.

**Methods:**

Randomized controlled trials (RCTs) were exclusively collected from PubMed, EMBASE, Web of Science, and The Cochrane Library, with a search deadline of April 30, 2023. Different exercise interventions on the physical performance of cancer patients were studied, and the Cochrane risk of bias assessment tool was employed to evaluate the quality of the included literature. Data analysis was conducted using STATA 15.1 software.

**Results:**

This study included ten randomized controlled trials with a combined sample size of 503 participants. Network meta-analysis results revealed that aerobic combined with resistance multimodal exercise could reduce fat mass in cancer patients (SUCRA: 92.3%). Resistance exercise could improve lean mass in cancer patients (SUCRA: 95.7%). Furthermore, resistance exercise could enhance leg extension functionality in cancer patients with sarcopenia (SUCRA: 83.0%).

**Conclusion:**

This study suggests that resistance exercise may be more beneficial for cancer-related sarcopenia.In clinical practice, exercise interventions should be tailored to the individual patients’ circumstances.

**Registration number:**

This review was registered on INPLASY2023110025; DOI number is 10.37766/inplasy2023.11.0025.

## Background

Sarcopenia is a systemic syndrome characterized primarily by the weakening or loss of muscle mass and function [1]. Its pathogenesis is associated with inflammatory responses, hormone levels, and insulin resistance within the body, subsequently leading to disruptions in protein synthesis and the onset of sarcopenia [2, 3]. Based on differing mechanisms of occurrence, sarcopenia is classified into primary (i.e., degenerative) and secondary types, with the former being more prevalent among older people. At the same time, the latter is commonly observed in cases of chronic wasting. Tumor patients constitute a prominent demographic affected by secondary muscle atrophy, with an incidence rate of approximately 38.6%. This condition is closely linked to postoperative complications, chemotherapy toxicity reactions, and overall survival rates [4]. Statistics reveal that for tumor patients, a 25% reduction in body mass corresponds to a loss of 75% of skeletal muscle myocardium protein. Even more concerning, sarcopenia is responsible for approximately one-fifth of all tumor-related fatalities [5–7].

So far, the existing drug treatments have not been entirely satisfactory, and the accompanying side effects and high medical costs have restricted the clinical application of such therapies. Therefore, the search for a cost-effective, low-side-effect, non-pharmaceutical alternative has become increasingly important. Several studies suggest that exercise interventions can effectively delay the onset of sarcopenia in cancer patients, improve their quality of life, and extend their survival periods, making them the most efficient measure for treating sarcopenia [8–11]. However, due to the diverse nature and distinct characteristics of exercise intervention measures, there is currently no unanimous consensus on which exercise intervention is the most effective.

Network meta-analysis is an advanced, evidence-based technique that enables direct or indirect comparisons of the effects of multiple interventions on a particular disease and ranks their relative efficacy for improvement [12]. In this study, we aim to evaluate the impact of various exercise interventions on the physical performance of cancer patients with sarcopenia. By comparing these exercise interventions, we aim to provide valuable insights for healthcare professionals and patients.

## Materials and methods

### Search strategy

Through a computer search encompassing four electronic databases, namely PubMed, EMBASE, Web of Science, and The Cochrane Library, covering the period from their inception to April 2023, the retrieval strategy was structured by the PICOS framework: (P) Population: cancer patients; (I) Intervention: exercise; (C) Control Group: a control group receiving standard care or stretching exercises exclusively; (O) Outcomes: lean mass, fat mass, and leg extension test (leg extension); (S) Study Type: randomized controlled trials. Taking PubMed as an example, the detailed search strategy is shown in Table 1.

**Table 1:** PubMed, with the search strategy as an example

#1	“Neoplasms” [Mesh]
#2	(((((((((((((((((“Neoplasms” [Mesh]) OR (Neoplasms[Title/Abstract])) OR (Tumor[Title/Abstract])) OR (Tumors[Title/Abstract])) OR (Neoplasia[Title/Abstract])) OR (Neoplasia[Title/Abstract])) OR (Cancer[Title/Abstract])) OR (Cancers[Title/Abstract])) OR (Malignant Neoplasm [Title/Abstract]))OR(Malignancy[Title/Abstract])) OR (Malignancies[Title/Abstract])) OR (Malignant Neoplasms[Title/Abstract])) OR (Neoplasm, Malignant[Title/Abstract])) OR (Neoplasms, Malignant[Title/Abstract])) OR (Benign Neoplasms[Title/Abstract])) OR (Benign Neoplasm[Title/Abstract])) OR (Neoplasms, Benign[Title/Abstract])) OR (Neoplasm, Benign[Title/Abstract])
#3	Sarcopenia” [Mesh]
#4	((“Sarcopenia” [Mesh]) OR (Sarcopenia)) OR (Sarcopenias)
#5	“Exercise” [Mesh]
#6	(((((((((((((((((((((((((“Exercise” [Mesh]) OR (exercise[Title/Abstract])) OR (Exercises[Title/Abstract])) OR (Physical Activity[Title/Abstract])) OR (Activities, Physical[Title/Abstract])) OR (Activity, Physical[Title/Abstract])) OR (Physical Activities[Title/Abstract])) OR (Exercise, Physical[Title/Abstract])) OR (Exercises, Physical[Title/Abstract])) OR (Physical Exercise[Title/Abstract])) OR (Physical Exercises[Title/Abstract])) OR (Acute Exercise[Title/Abstract])) ) OR (Exercises, Acute[Title/Abstract])) OR (Exercise, Isometric[Title/Abstract])) OR (Exercises, Isometric[Title/Abstract])) OR (Isometric Exercises[Title/Abstract])) OR (Isometric Exercise[Title/Abstract])) OR (Exercise, Aerobic[Title/Abstract]))OR (Aerobic Exercise[Title/Abstract])) OR (Aerobic Exercises[Title/Abstract])) OR (Exercises, Aerobic[Title/Abstract])) OR (Exercise Training[Title/Abstract])) OR (Exercise Trainings[Title/Abstract])) OR (Training, Exercise[Title/Abstract])) OR (Trainings, Exercise[Title/Abstract])
#7	(#2)AND(#4)AND(#6)

### Inclusion criteria

(1) The experimental group and various exercise training methods as interventions for tumor patients. (2) The control group comprises patients receiving exercise interventions distinct from those in the experimental group or receiving routine care. (3) A clinical randomized controlled trial. (4) Outcomes encompass at least one of the following indicators: lean mass, fat mass, and leg extension test.

### Exclusion criteria

(1) Literature lacking complete or accessible data; (2) non-randomized controlled trials, including quasi-randomized controlled trials and animal studies; (3) conference abstracts, case reports, and communications; (4) outcome measures that cannot be converted or aggregated; (5) literature not in the English language.

### Study selection

Literature screening and exclusion were carried out using EndNote 20, a literature management software. Initially, two researchers independently conducted literature screening using the inclusion and exclusion criteria. Duplicate titles, non-randomized controlled trial studies, retrospective papers, conference papers, protocols, and correspondence were eliminated. Subsequently, the abstracts of the remaining literature were reviewed to determine their inclusion or exclusion. Any remaining literature was then subjected to a cross-check and comparison by both researchers. If the assessments were identical, the literature was included; in cases of disagreement, the third investigator facilitated discussion and resolution.

### Data extraction

Seven predetermined data elements were chosen: (1) author’s name, (2) year of publication, (3) country, (4) study duration, (5) sample size, (6) mean age, and (7) outcome measures for exercise intervention.

### Literature quality evaluation

The assessment of literature quality was conducted independently by two researchers, with a subsequent thorough review of the results. In cases of disagreement, a third party was consulted for evaluation. The evaluation of the risk of bias was carried out using the Cochrane 5.1.0 Risk Assessment Tool (ROB), considering seven key domains: (1) random sequence generation; (2) allocation concealment; (3) blinding of participants and experimenters; (4) investigator blinding; (5) completeness of outcome data; (6) selective reporting of results; and (7) other potential sources of bias. Each domain was categorized as having “high risk of bias,” “low risk of bias,” or “unclear.” Trials were then stratified into three levels of risk of bias based on the number of components with high ROB: high risk (5 or more), moderate risk (3 to 4), and low risk (2 or fewer) [13]. The results were ultimately presented in charts and tables.

### Data analysis

Employing various exercise interventions, all outcome measures were treated as continuous variables, and the presentation and analysis included mean, standard deviation (SD) and mean-variance (MD, representing the absolute difference between the treatment and control groups and calculated using the same sample size) or standardized mean difference (SMD, indicating the mean of the groups divided by the standard deviation between subjects, suitable for data analysis in trials of varying sizes), along with 95% confidence interval (CI) [14]. Given the heterogeneity among studies, we opted for a random effects model for the analysis rather than a fixed effects model [15].

The Stata software, version 15.1, was employed for the NMA summary and analysis, utilizing the Bayesian Markov Chain Monte Carlo algorithm. To assess consistency, node splitting was applied, with a threshold of a *p*-value greater than 0.05 indicating the use of the consistency model; otherwise, the inconsistency model was employed [16]. Stata generated the network graph, where each node represents an independent intervention, and the connecting lines between nodes signify direct comparisons between interventions. The size of each node and the width of the lines are proportional to the number of trials conducted [17].

The greater the SUCRA value, the higher the likelihood of being the most effective intervention [17]. When determining the ranking of SUCRA, in addition to comparing the area under the cumulative ranking probability curve for different exercise interventions (surface under the cumulative ranking, SUCRA), it is essential to interpret the clinical significance of these interventions carefully. Furthermore, to address the possibility of publication bias in NMA, we constructed a network funnel plot and visually assessed its symmetry to detect the presence of small-sample effects [17].

## Results

### Literature screening process

**Fig. 1:**
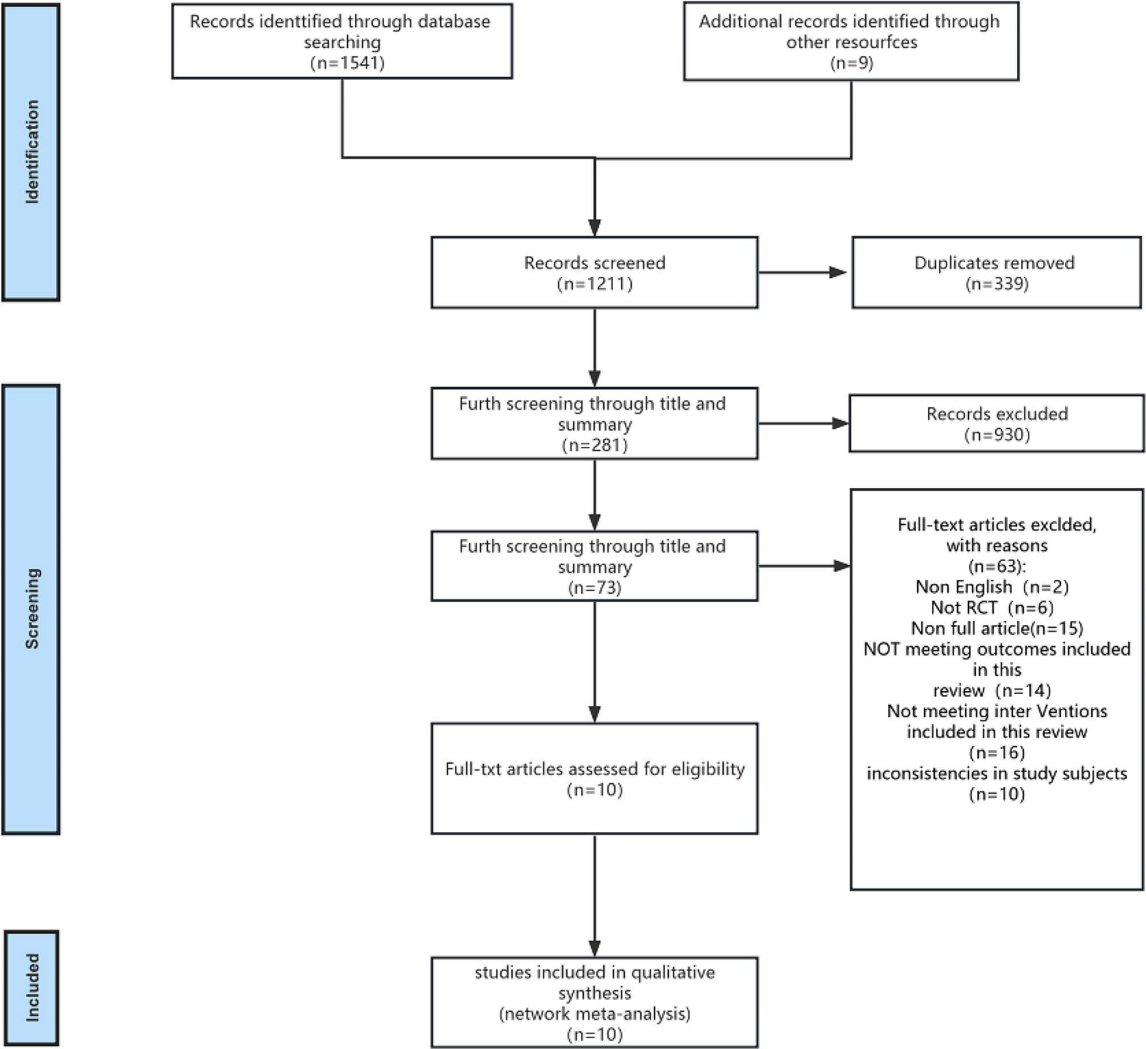
Flow chart of literature screening

Following a thorough search across multiple databases, an initial screening identified 1,541 relevant articles. A manual search yielded nine more articles, and with the assistance of Endnote software, 339 duplicate entries were removed. Subsequent examinations of titles and abstracts resulted in excluding 1,106 articles deemed irrelevant. This process left 73 articles for full-text evaluation, eventually culminating in the inclusion of 10 pieces in the meta-analysis (Fig. 1).

### Literature quality evaluation

**Fig. 2:**
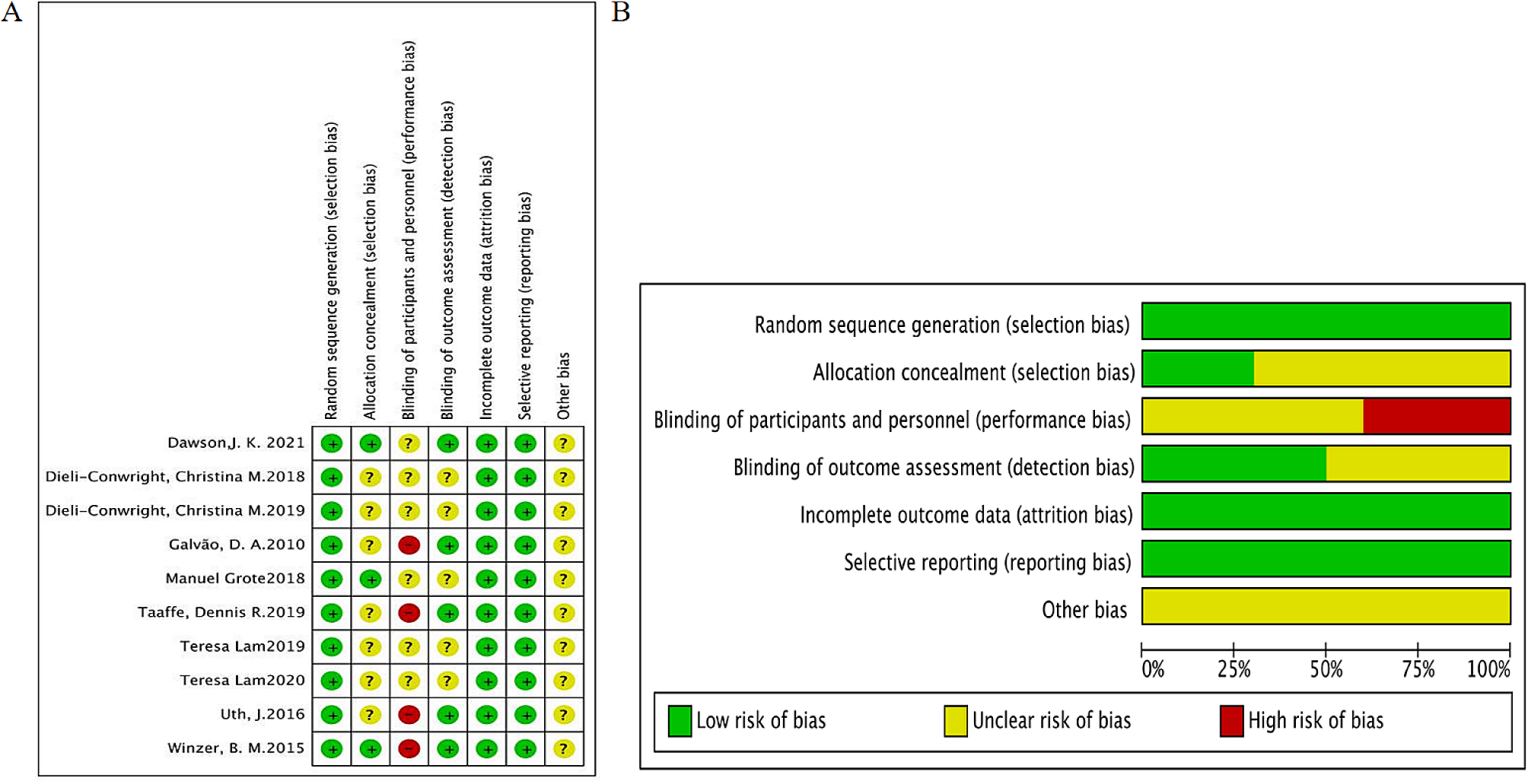
(**A**) Risk of bias plot for literature quality assessment; (**B**) Scale plot of risk of bias for literature quality evaluation

Out of the ten studies included [11, 18–26], 2 of them [11, 22] were categorized as low risk, while 8 [11, 20, 22, 25] were classified as medium risk. All the included studies referred to random allocation, with 4 [11, 18, 20, 21] explicitly mentioning the use of computerized grouping, while the remaining literature indicated randomization. In three [11, 21, 22] of the studies, a specific concealed allocation scheme was proposed. Due to the nature of the exercise intervention, achieving blinding for both the subjects and assessors was challenging, as patients and their families needed to provide informed consent before participating in the experiments. All the studies described the rate and reasons for loss to follow-up, and the outcome measures were comprehensive. The baseline characteristics of the intervention groups were reasonably balanced, with no signs of selective reporting. For detailed information, please refer to Fig. 2A and B.

### Basic characteristics of the included literature

This study incorporated ten randomized controlled trials (RCTs), encompassing 503 patients diagnosed with malignancies, with 310 male and 193 female participants. The selected studies contained four distinct types of exercises models: 5 RCTs introduced resistance exercises in the experimental group [11, 19, 21, 25], 5 RCTs implemented aerobic combined with resistance multimodal exercise [16, 18, 22–24], and 2 RCTs within the control group utilized stretching exercises [11, 22], while the remaining control groups involved routine activities without any additional interventions. All ten of the included studies reported fat mass as an outcome measure [11, 18–26], lean mass as an outcome measure [11, 18–26], and six studies measured leg extension as an outcome indicator [11, 18–22]. These studies were distributed across regions, with three originating from the Americas, two from Europe, and six from Oceania. Detailed characteristics of the included studies are presented in Table 2.

**Table 2:** Characteristics of included studies

Author	Country	Year	Population	Age	Total/male/female	Intervention	Control	Outcome
Dawson, J. K.	America	2021	Androgen deprivation therapy in prostate cancer	T:68.6 ± 8.4 C:66.3 ± 9.0	T:12/2/10 C:12/9/3	Resistance training Length of Intervention: 12 weeks Freq: 3 times a week Duration: 50 min Intensity: Appropriate resistance	Stretching exercise	Fat mass Lass mas Leg extension (kgs)
Dieli-Conwri ght	America	2018	breast cancer	T: 53 6( 10.4) C: 53.6 ( 10. 1)	T: 25/15/7 C: 26/17/9	Aerobic and resistance exercise Length of Intervention: 16 weeks Freq: 3 times a week Duration: 50–80 min Intensity: moderate to vigorous 65% to 85% of heart rate maximum	Routine	Fat mass Lass mas Leg extension (kgs)
Dieli-Conwri ght	America	2019	breast cancer	T: 65.99(4.3) C: 66(8.55)	T: 15/11/4 C: 15/12/3	Aerobic and resistance exercise Length of Intervention: 16 weeks Freq: 3 times a week Duration: 50–80 min intensity: moderate to vigorous 65 to 85% of heart rate maximum	Routine	Fat mass Lass mas
Galvão, D. A.	Australia	2010	Prostate cancer undergoing AST	T: 69.5(7.3) C: 70. 1(7.3)	T: 13/11/2 C: 14/11/3	Resistance exercise Length of Intervention: 12 weeks Freq: 2 a week Duration: 40–50 min Intensity: 65 to 80% maximum heart rate	Routine	Fat mass Lass mas Leg extension (kgs)
Grote Manuel	Germany	2018	head and neck cancer	T: 60.2 (4.7) C: 61.5( 15.7)	T: 25/12/ 13 C: 24/13/ 11	Resistance exercise Length of Intervention: 5 weeks Freq: 5 times a week Duration: 30 min Intensity: -	Routine	Fat mass Lass mas
Teresa Lam	Australia and New Zealand	2019	Androgen deprivation therapy in prostate cancer	T: 69.3(2.3) C: 71.7 ( 1.9)	T: 28/12/16 C: 25/5/20	Resistance exercise Length of Intervention: 6 weeks Freq: 3 times a week Duration: three sets per exercise with 8– 12 repetitions Intensity: The exercise or the loading was advanced with strength adaptation	Routine	Fat mass Lass mas Leg extension (kgs)
Teresa Lam	Australia	2020	Androgen deprivation therapy in prostate cancer	T: 69.3(2.3) C: 71.7 ( 1.9)	T: 28/12/16 C: 25/5/20	Resistance exercise Length of Intervention: 56 weeks Freq: three times a week Duration: three sets per exercise with 8– 12 repetitions Intensity: The exercise or the loading was advanced with strength adaptation	Routine	Fat mass Lass mas Leg extension (kgs)
Uth, J.	Denmark	2016	Androgen deprivation Therapy in prostate cancer	T: 67. 1(7. 1) C: 66.5(4.9)	T: 10/5/5 C: 10/5/5	Aerobic and resistance exercise Length of Intervention:32 weeks Freq:5 times a week Duration: 45–60 min Intensity: -	Routine	Fat mass lass mass Leg extension (kgs)
Winzer, B. M	Australia	2015	Esophageal adenocarcino ma	T: 57.2 (7.5) C: 57.6( 10.4)	T: 40/24/ 16 C: 40/27/ 13	Aerobic and resistance exercise Length of Intervention: 24 weeks Freq: 3 times a week Duration: 60 min Intensity: moderate	Stretching exercise	Fat mass Lass mas
Taaffe Denni’s R	Australia	2019	Androgen deprivation therapy in prostate cancer	T: 57.2 (7.5) C: 57.6( 10.4)	T: 12/3/3 C: 12/4/2	Aerobic and resistance exercise Length of Intervention: 56 weeks Freq: 3 times a week Duration: 60 min Intensity: 60–85% estimated maximum heart rate	Routine	Fat mass Lass mass

Note: Routine: routine care (no exercise)

### Mesh Meta-analysis results

**Fig. 3:**
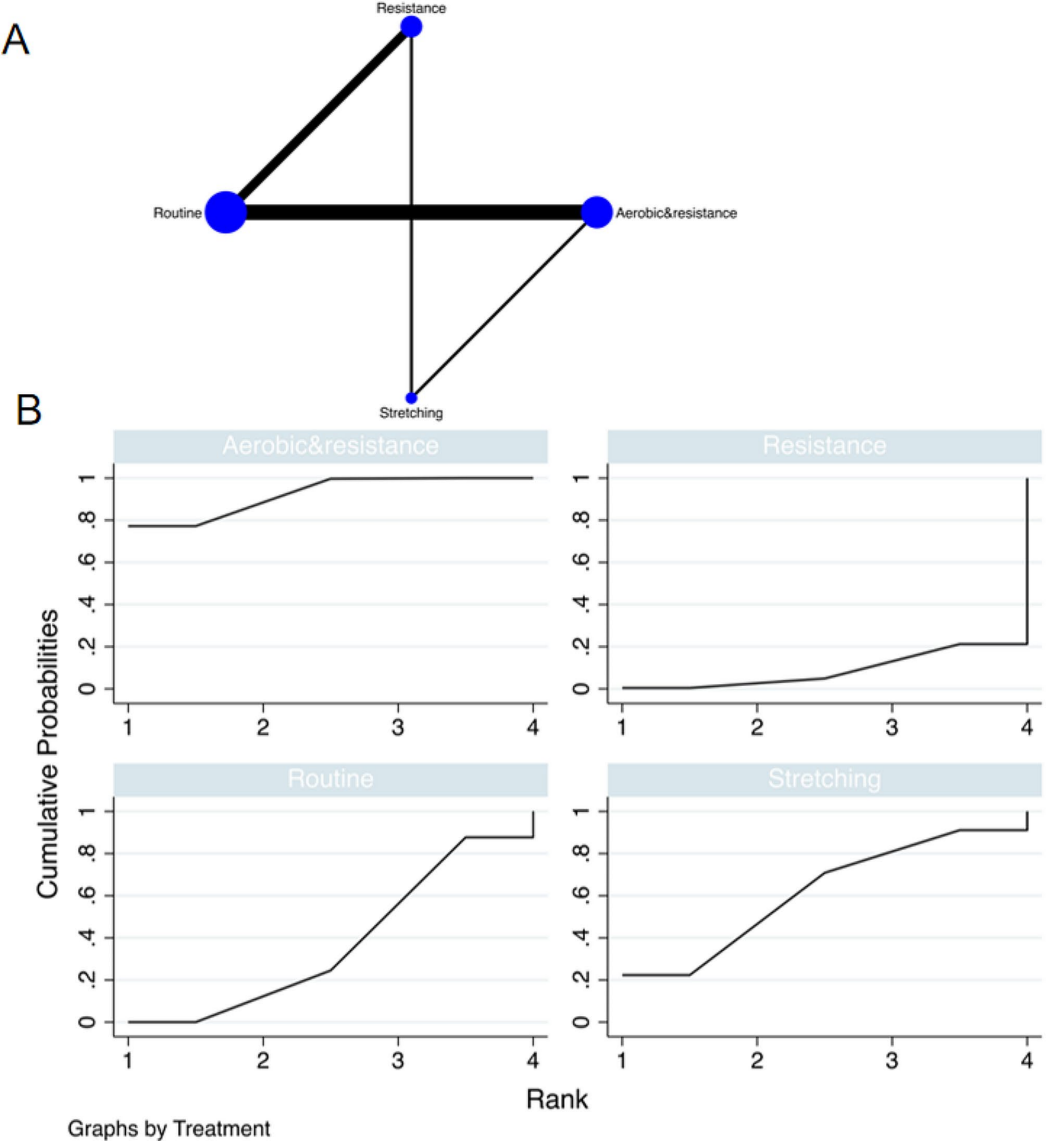
The NMA plot of fat mass; B Fat mass SUCRA Fig

**Fig. 4:**
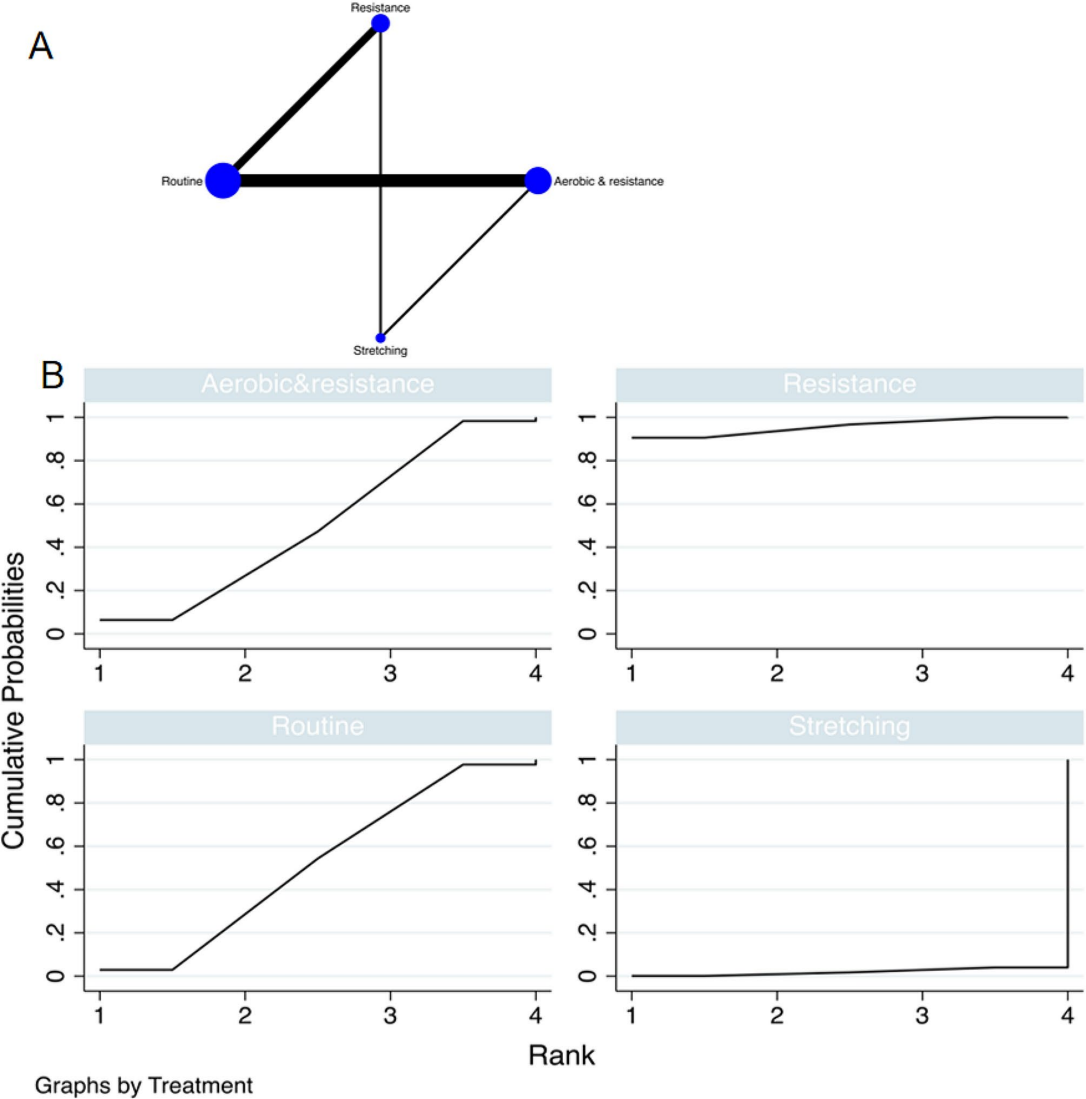
The NMA plot of lean mass; B Lean mass SUCRA Fig

**Fig. 5:**
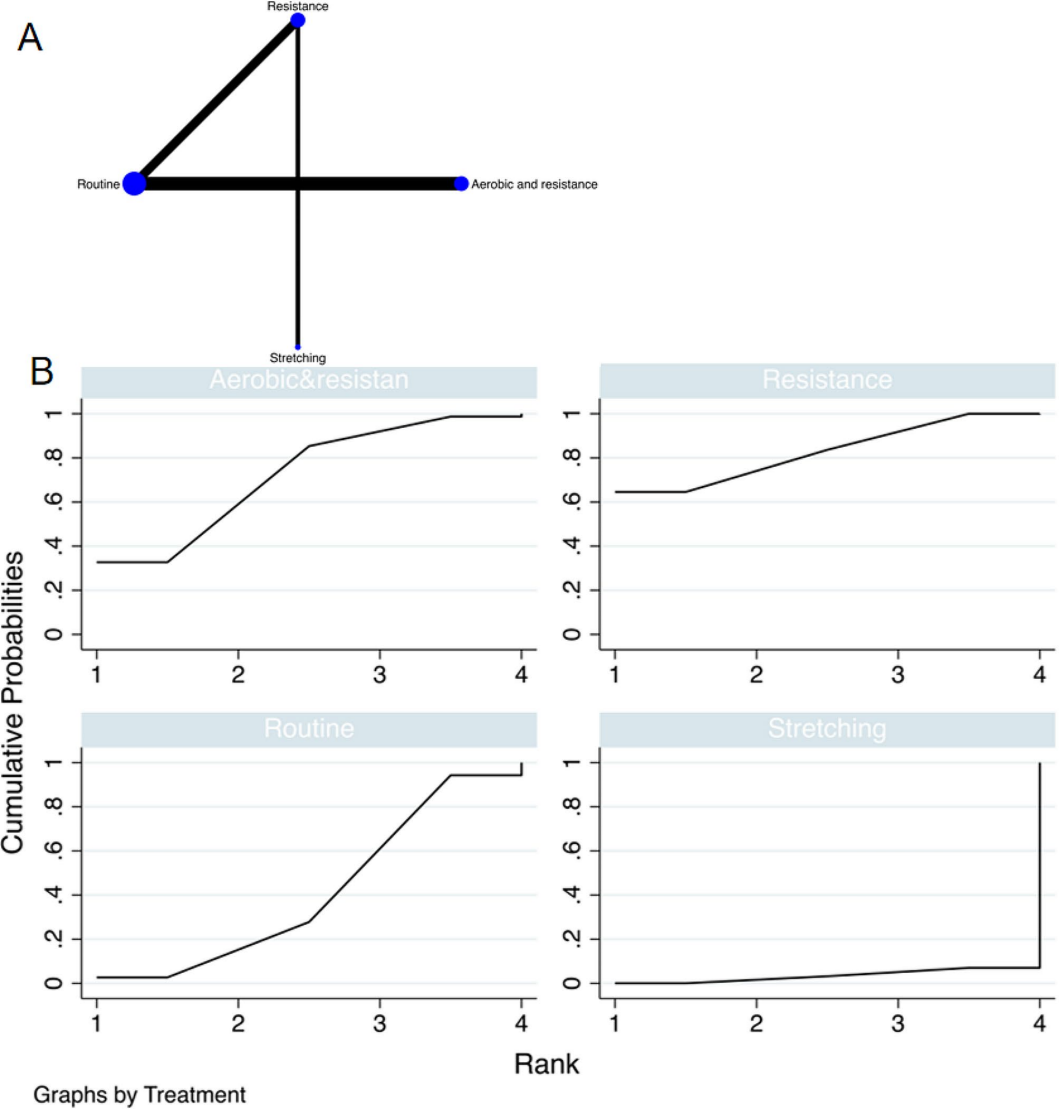
The NMA plot of leg extension; B Leg extension SUCRA Fig

The full figures are detailed in Figs. 3A and 4A, and 5A.

#### Fat mass

A total of 10 randomized controlled trials (RCTs) was included, and multiple exercise interventions, primarily focusing on routine exercise, formed a complex network structure comprising two interconnected loops. The node-splitting test assessed the consistency between indirect and direct outcomes from all studies. The results of the network meta-analysis indicated that stretching [MD = -4.02, 95% CI = (10.42, 2.38)] and aerobic combined with resistance multi modal exercise [MD = -6.09, 95% CI = (-10.84, -1.34)] exhibited superior performance compared to conventional practice in comparison with the control group. The top-ranking intervention, based on the best-ranking results, was aerobic combined with resistance multimodal exercise, demonstrating the most effective reduction in tumor fat mass (SUCRA: 92.3%, as depicted in Fig. 3B). Further comparisons between different exercise interventions are presented in Table 3.

**Table 3:** Fat mass League diagram

Aerobic & resistance	Stretching	Routine	Resistance
Aerobic & resistance	2.07 (-3.22,7.36)	3.82 ( 1.60,6.03)	6.09 ( 1.34, 10.84)
-2.07 (-7.36,3.22)	Stretching	1.74 (-3.85,7.33)	4.02 (-2.38, 10.42)
-3.82 (-6.03,- 1.60)	− 1.74 (-7.33,3.85)	Routine	2.27 (-2.08,6.63)
-6.09 (− 10.84,- 1.34)	-4.02 (− 10.42,2.38)	-2.27 (-6.63,2.08)	Resistance

#### Lean mass

A total of 10 randomized controlled trials (RCTs) were included in the analysis. Multiple exercise interventions, predominantly centered around routine exercises, formed a complex network structure, resulting in two interconnected closed loops. Consistency between indirect and direct outcome indicators from all studies was evaluated. The network meta-analysis revealed that resistance exercise [MD = 14.00, 95% CI = (4.41, 23.60)] outperformed conventional exercise compared to the control group. The highest-ranking results demonstrated that resistance exercise was the most effective in increasing lean mass in tumor patients with sarcopenia (SUCRA: 95.7%, as depicted in Fig. 4B). Detailed comparisons between the various exercise interventions can be found in Table 4.

**Table 4:** lean mass League diagram

Resistance	Routine	Aerobic & resistance	Stretching
Resistance	-5.10 (− 11.24, 1.04)	-5.30 (− 12.48, 1.87)	− 14.00 (-23.60,-4.41)
5.10 (− 1.04, 11.24)	Routine	-0.20 (-4.45,4.05)	-8.90 (− 18.21,0.41)
5.30 (− 1.87, 12.48)	0.20 (-4.05,4.45)	Aerobic & resistance	-8.70 (− 17.67,0.28)
14.00 (4.41,23.60)	8.90 (-0.41, 18.21)	8.70 (-0.28, 17.67)	Stretching

#### Leg extension

A total of 6 randomized controlled trials (RCTs) were incorporated into the study, and various exercise interventions were primarily based on routine exercises, resulting in a complex network structure with a closed loop. To assess the consistency between indirect and direct indicators from all studies, the node-splitting test was employed. The network meta-analysis indicated that resistance exercise [MD = 68.27, 95% CI = (26.25, 110.30)] and aerobic combined with resistance multimodal exercise [MD = 57.97, 95% CI = (5.23, 121.16)] outperformed the control group. The top-ranking results highlighted that resistance exercise was the most effective in enhancing the Leg extension function in cancer patients with sarcopenia (SUCRA: 83.0%, as displayed in Fig. 5B). Detailed comparisons between the diverse exercise interventions can be found in Table 5.

**Table 5:** Leg extension League diagram

Resistance	Aerobic & resistance	Routine	Stretching
Resistance	− 10.31 (-58.32,37.70)	-21.90 (-66.31,22.51)	-68.27 (− 110.30,-26.25)
10.31 (-37.70,58.32)	Aerobic & resistance	− 11.59 (-32.92,9.73)	-57.97 (− 121.16,5.23)
21.90 (-22.51,66.31)	11.59 (-9.73,32.92)	Routine	-46.37 (− 106.98, 14.24)
68.27 (26.25, 110.30)	57.97 (-5.23, 121.16)	46.37 (− 14.24, 106.98)	Stretching

### Publication bias trial

**Fig. 6:**
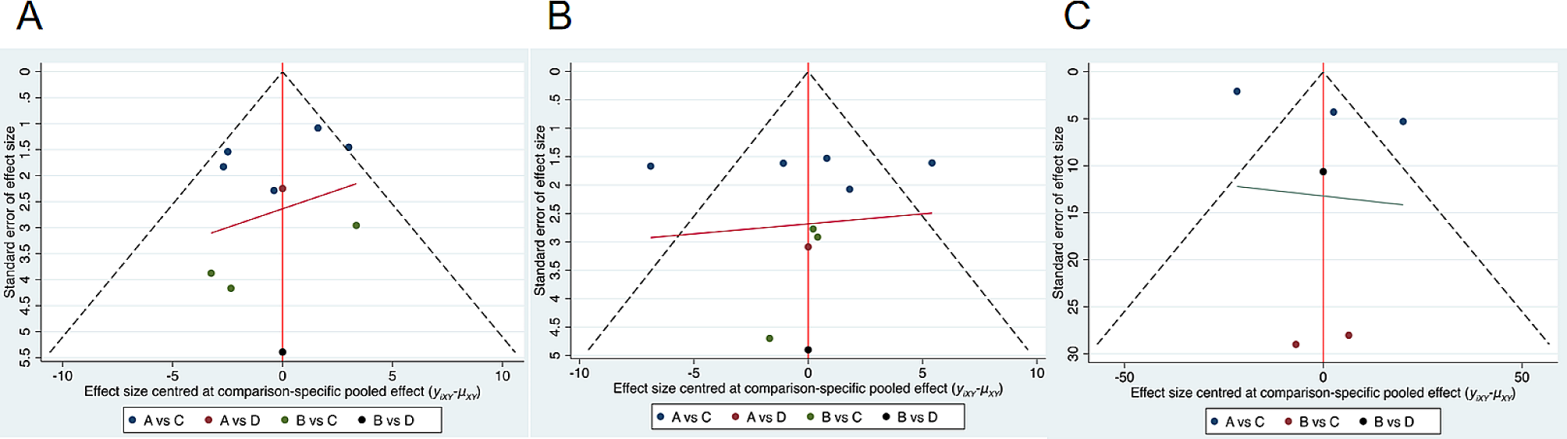
(**A**) The funnel plot of fat mass bias; (**B**) The funnel plot of lean mass bias; (**C**) The funnel plot of leg extension bias

A publication bias funnel plot was generated for the intervention effect indicators of various exercise modalities on sarcopenia in cancer patients (Fig. 6A, B and C), and no notable publication bias was observed upon visual inspection of the funnel plot.

## Discussion

A total of 10 randomized controlled trials (RCTs) were included, comprising a combined total of 503 cancer patients. Through the comparative analysis of the effects of various exercise interventions, including aerobic exercise, resistance exercise, aerobic resistance combined with multimodal training, and stretching exercise in cancer patients, our study demonstrates that differences in exercise interventions highlight the variability in their capacity to enhance sarcopenia and function in patients with sarcopenia-related tumours. Cancer patients must select targeted exercise interventions carefully. Resistance exercise exhibits the most favourable impact on improving lean mass and leg extension, while aerobic combined with resistance multimodal exercise is most effective in reducing fat mass in tumor patients with sarcopenia. Upon a comprehensive assessment, we assert that resistance exercise is competitive in ameliorating sarcopenia in cancer patients.

While ‘sarcopenia’ is defined as the loss of muscle mass or function, it is increasingly acknowledged that sarcopenia can also coexist with obesity. Excessive fat can obscure the absence of skeletal muscle mass. Thus, the measurement of fat mass is an essential component in the diagnosis of sarcopenia. Early detection, diagnosis, and exercise intervention are pivotal for prognosis improvement and sarcopenia treatment [27–29]. For cancer patients, engaging in 150 min of moderate or 75 min of high-intensity exercise per week is deemed safe. It can reduce abnormal lipid accumulation in skeletal muscle, enhance glucose circulation metabolism, and decrease fat mass [18, 30–32]. Regular fat mass measurements enable dynamic patient monitoring and adjustment of the regimen to achieve the optimal treatment outcome. The results demonstrate that combined resistance exercise is effective in reducing fat mass, which is statistically significant when compared to the control group. In contrast to resistance exercise, aerobic combined with resistance exercise offers more diversity, not only increasing exercise engagement but also boosting participants’ motivation, thus yielding more pronounced exercise effects.

Within the domain of studies about “sarcopenia,” assessments of lean mass (also known as lean weight or fat-free mass) are customarily incorporated, as they represent one of the paramount indicators of sarcopenia [31]. The reduction in muscle mass observed in sarcopenia often stems from a combination of muscle atrophy and cell death. At the molecular level, previous research has pointed to the association between sarcopenia and mitochondrial dysfunction, along with alterations in protein synthesis and degradation [32–34]. The measurement of lean mass allows us to furnish concrete evidence regarding the effectiveness of exercise interventions on skeletal muscle. Exercise interventions have the potential to stimulate the production of crucial regulatory components within skeletal muscle mitochondria, suppress ubiquitin-proteasome system (UPS) activity, enhance the expression of autophagy-related genes, improve mitochondrial oxidative capacity, and increase muscle blood flow [35–37]. Our findings indicate that resistance exercise yields significant intervention effects on lean mass in patients with tumor-related sarcopenia. This can be attributed to the stimulation of mitochondrial “biogenesis” through resistance exercise, which accelerates muscle cell signalling, increases mitochondrial count, enhances glucose transporter capacity and reduces the production of muscle growth inhibitors. Consequently, this process inhibits the proliferation and differentiation of myoblasts in developing muscles [38–40].

The relationship between lower limb strength and physical function is more closely intertwined than that of the upper limbs. Resistance exercise has found widespread use in the treatment of tumor-related sarcopenia patients. Impedance exercise, involving the application of external force to facilitate synergistic and antagonistic muscle training, can enhance lower limb muscle strength and endurance among patients with tumor-related sarcopenia, thus leading to improved leg extension function. Our results demonstrate a significant intervention effect of resistance exercise on leg extension function in tumor-related sarcopenia patients. This can be attributed to the requirements of muscle strength during resistance exercise, which consequently impacts the power of muscle motor units and the number and type of muscle fibers and fosters muscle growth and repair. Such improvements in muscle contraction afford patients greater control during leg extension, reducing muscle fatigue and pain [38–40]. Furthermore, the persistent mechanical strain on osteocytes induced by resistance training can promote their physical deformation, subsequently expediting bone remodeling and tissue regeneration and ultimately enhancing bone density [41]. Therefore, the study of tumor-related sarcopenia patients underscores that regular resistance exercise training represents a practical approach for restoring and enhancing leg extension function, ultimately improving the overall quality of life.

## Advantage and limitations

Therapies aimed at addressing tumor-induced sarcopenia have garnered significant attention. Moderate exercise has demonstrated its potential to enhance overall bodily function and augment muscle mass in tumor patients. This study consolidates data from 10 eligible studies involving 503 patients to corroborate the efficacy of four exercise modes: resistance exercise, aerobic combined resistance multimodal exercise, and stretching exercise. In comparison to the meta-analysis of relevant literature, resistance exercise has shown a more pronounced impact on patients by bolstering both muscle strength and mass. This study provides valuable guidance for future research endeavors.

This study has its limitations. First, it relies on currently available English literature about tumor sarcopenia, which may introduce certain constraints to the study findings. Second, there exists some degree of heterogeneity and bias in the study results. Third, the research is influenced by variations in disease types, cultural backgrounds, and the healthcare systems of cancer patients. Therefore, it is imperative to tailor exercise interventions to individual needs based on the specific circumstances of each patient. Our study has not yet included aerobic capacity testing and leg press testing, and thus cannot assess physical performance, which is what we are working towards in the future.

## Conclusion

The findings of this study underscore the superiority of resistance exercise over other exercise modalities in enhancing muscle mass and function. Consequently, the inclusion of resistance exercise in the rehabilitation regimen for cancer patients is of paramount importance. Tailoring the intervention to the individual circumstances of the patients and implementing it promptly is crucial for achieving optimal outcomes. Additionally, further investigation into the specific application methods and results of exercise in the rehabilitation of cancer patients is warranted to offer more scientifically informed guidance for clinical practice.

## Data Availability

The datasets used and/or analysis during the current study are available from the corresponding author on reasonable request.

## Abbreviations

RCTs: Randomized controlled trials
ROB: Risk Assessment Tool
SD: Standard deviation
CI: Confidence interval
